# Age- and Risk-Based Stratification in Dyspepsia: Redefining Endoscopic Thresholds for Clinically Significant and Malignant Findings

**DOI:** 10.3390/clinpract16010007

**Published:** 2025-12-30

**Authors:** Oren Gal, Dorin Nicola, Amir Mari, Randa Natour, Noor Fanadka, Ahlam Bsoul, Ahmad Mahamid, Rawi Hazzan, Fadi Abu Baker

**Affiliations:** 1 Department of Gastroenterology and Hepatology, Hillel Yaffe Medical Center, Hadera 38100, Israel; 2 Department of Gastroenterology, Nazareth EMMS Hospital, Affiliated with the Faculty of Medicine, Bar-Ilan University, Safed 52900, Israel; 3 Department of Surgery, Carmel Medical Center, Haifa 34361, Israel; 4 Bruce Rappaport Faculty of Medicine, Technion–Israel Institute of Technology, Haifa 32000, Israel; 5 Liver Clinic, Clalit Health Service—Northern District, Nazareth 19132, Israel; 6 Azrieli Faculty of Medicine, Bar-Ilan University, Safed 52900, Israel

**Keywords:** alarm symptoms, clinically significant findings, functional dyspepsia, gastroscopy, outcomes

## Abstract

Background: Dyspepsia is a common indication for gastroscopy, yet its diagnostic yield for malignancy and other clinically significant findings (CSF) remains low. Improved risk stratification is therefore essential to guide endoscopic referral. This study evaluates the diagnostic yield of gastroscopy in dyspepsia and investigates the predictive roles of age, ethnicity, and alarm symptoms. Methods: This retrospective single-center study was conducted at a university-affiliated hospital in Israel and included 3022 patients who underwent gastroscopy for dyspepsia over a five-year period. Multivariate logistic regression identified independent predictors of CSF, and receiver operating characteristic (ROC) analysis determined optimal age thresholds for malignancy and CSF. Results: Functional dyspepsia accounted for 55.9% of cases, while precancerous gastric lesions and upper gastrointestinal malignancies were identified in 12.8% and 0.79%, respectively. In multivariable models, age ≥ 50 years (OR = 2.59; CI: 2.02–3.32) and alarm symptoms (OR = 1.79; 95% CI: 1.33–2.41) independently predicted CSFs. Malignancy was similarly associated with age ≥ 50 years (OR = 4.89; CI: 1.11–21.60) and alarm symptoms (OR = 31.42; CI: 10.26–96.19). ROC analysis identified optimal age thresholds of 50 years for CSF (AUC = 0.65) and 54 years for malignancy (AUC = 0.72). Ethnicity did not independently predict malignancy, though minority patients showed differing precancerous lesion patterns. Conclusions: Age ≥ 50 years and alarm symptoms significantly increased the likelihood of CSFs and malignancy, supporting a selective approach to gastroscopy. ROC-derived thresholds may support reconsideration of age criteria in settings with similar epidemiologic patterns, highlighting the need for region-specific risk stratification.

## 1. Introduction

Dyspepsia is a highly prevalent clinical syndrome, with estimates of up to 40% in Western populations [1,2]. Prevalence varies across studies due to differences in diagnostic criteria, geographic distribution, sex, and age. Dyspeptic symptoms—including epigastric pain or burning, postprandial fullness, early satiety, bloating, and belching—frequently overlap with other upper gastrointestinal (GI) disorders such as gastroesophageal reflux disease (GERD) [3], contributing to diagnostic complexity and heterogeneity in clinical presentation.

Functional dyspepsia (FD) is defined by persistent or recurrent epigastric symptoms in the absence of structural disease on appropriate evaluation, including gastroscopy [4,5]. According to the Rome criteria, FD encompasses two primary phenotypes: epigastric pain syndrome (EPS), characterized by epigastric pain or burning unrelated to meals, and postprandial distress syndrome (PDS), defined by postprandial fullness and early satiety [6,7,8].

The pathophysiology of FD is multifactorial, reflecting abnormalities in gastric sensory–motor function, impaired fundic accommodation, delayed gastric emptying, visceral hypersensitivity, alterations in central pain modulation, and duodenal mucosal abnormalities such as increased permeability, low-grade inflammation, and eosinophil–mast cell activation [9,10,11,12,13].

From an epidemiological standpoint, dyspepsia encompasses a spectrum of underlying conditions While peptic ulcer disease and gastritis, most commonly related to *Helicobacter pylori* (*H. pylori*) infection or NSAID use, constitute the principal organic etiologies [14,15], the majority of patients undergoing upper GI endoscopy are ultimately classified as having FD [16]. Conversely, upper GI malignancies constitute a rare cause of dyspepsia in Western populations, though risk increases with age and varies markedly across geographic regions, with substantially higher incidence in parts of Asia [17]. These regional differences underscore the need for tailored approaches to evaluating dyspeptic patients.

Endoscopy guidelines have evolved in response to the high prevalence of FD, the generally low diagnostic yield of gastroscopy, and the disproportionate healthcare utilization associated with early invasive testing. Earlier recommendations advocated endoscopy for patients ≥55 years or those presenting with alarm features [18], whereas the most recent American College of Gastroenterology and Canadian Association of Gastroenterology (ACG/CAG) guidelines increased the age threshold to 60 years and deemphasized routine use of alarm features owing to their limited predictive value for malignancy [19,20]. Consequently, endoscopic referral thresholds must be interpreted within the context of local malignancy rates, population demographics, and healthcare system characteristics.

While global patterns of dyspepsia, endoscopic yield, and FD epidemiology have been described extensively [21,22,23], region-specific data remain scarce, despite substantial ethnic diversity and known differences in *H. pylori* prevalence and gastric pathology across populations. Local evidence is particularly important for informing risk-stratified endoscopy strategies, given the interplay between age, ethnicity, alarm symptoms, and underlying disease prevalence.

Against this background, this study aimed to characterize the diagnostic yield of gastroscopy in a large, ethnically diverse cohort of dyspeptic patients in Israel. The primary objective was to determine the prevalence and predictors of upper gastrointestinal malignancy and other clinically significant findings (CSFs). Secondary objectives included assessing the burden of precancerous gastric conditions and evaluating the predictive roles of age, sex, ethnicity, and alarm symptoms. By integrating clinical, endoscopic, and histologic data with receiver operating characteristic (ROC)–derived age thresholds, this study aims to refine region-specific risk stratification and support more judicious use of gastroscopy in the evaluation of dyspepsia.

## 2. Materials and Methods

### 2.1. Study Design and Population

This retrospective, single-center cohort study was conducted by reviewing electronic medical records of patients who underwent gastroscopy for dyspepsia over a five-year period (2018–2023) at the Gastroenterology Department of Hillel Yaffe Medical Center, a university-affiliated hospital in Israel. Patient data were retrieved from the department’s electronic medical record system. Dyspepsia was defined by the presence of postprandial fullness, early satiety, epigastric pain, or burning. Endoscopy reports were systematically reviewed to identify patients referred for endoscopic evaluation due to dyspeptic symptoms, and both endoscopic and histologic findings were analyzed. Patients were referred predominantly from community primary care and gastroenterology outpatient clinics, reflecting the typical elective pathway for dyspepsia evaluation in our healthcare setting.

Only patients with complete clinical and endoscopic data who were undergoing gastroscopy for an initial (index) evaluation of dyspepsia were included. Patients were excluded if they were younger than 18 years; had a history of upper gastrointestinal surgery (including bariatric procedures) or cholecystectomy; had undergone any prior upper endoscopy; or had previously documented dyspepsia, PUD, erosive esophagitis, or large hiatal hernia. These criteria ensured that the cohort represented first-time evaluations consistent with the clinical scenarios addressed in dyspepsia guidelines. Symptom duration (new-onset vs. chronic dyspepsia) was not consistently recorded in the electronic medical record and therefore could not be analyzed.

The study was approved by the Institutional Review Board of Hillel Yaffe Medical Center (protocol code HYMC-0010-24). Given its retrospective nature, an exemption from informed consent was granted, as data collection did not influence patient management, and all individuals received standard medical care.

### 2.2. Data Collection and Classification

Demographic variables, including age, sex, ethnicity, and place of birth, were recorded at the time of hospital admission based on a national database. Ethnicity was classified according to the Israeli Central Bureau of Statistics into two primary groups: Arabs and Jews, with Arabs constituting approximately one-fifth of the national population.

Clinical indications for gastroscopy were categorized as dyspepsia alone or dyspepsia associated with alarm symptoms, which included dysphagia, weight loss, persistent vomiting, or iron-deficiency anemia (IDA). IDA was defined as hemoglobin < 13 g/dL in men and <12 g/dL in women, in combination with a serum ferritin < 30 ng/mL. Endoscopic and histologic findings were systematically recorded, allowing for an assessment of the etiologic distribution of dyspepsia and the prevalence of FD. Endoscopic gastritis was documented according to the endoscopist’s visual assessment and should be interpreted with caution, as visual mucosal changes may overestimate the presence or severity of histologic gastritis. When biopsies were obtained, this was performed at the discretion of the endoscopist and, in our institution, typically followed the principles of the updated Sydney protocol in cases of suspected gastritis, PUD, or premalignant gastric changes, with targeted sampling from the antrum, incisura angularis, and corpus. Specimens were processed with routine hematoxylin–eosin staining, and Giemsa stain was used when needed to identify *H. pylori*. Atrophic gastritis and intestinal metaplasia were classified according to the updated Sydney System, whereas gastric epithelial dysplasia was diagnosed and graded by expert gastrointestinal pathologists in accordance with the 2019 World Health Organization (WHO) criteria. These histopathologic definitions were applied to identify precancerous gastric conditions relevant to the study’s main outcomes.

FD was defined as dyspeptic symptoms in the absence of clinically significant endoscopic or histologic abnormalities. Patients were classified as having FD if they demonstrated normal gastroscopy findings or presented with mild gastritis or duodenitis without evidence of *H. pylori* infection. Small hiatus hernias and reactive or nonspecific histologic findings were also included within this category, provided that no evidence of gastric or esophageal cancer, reflux esophagitis, large or paraesophageal hiatus hernia, Barrett’s esophagus, or moderate-to-severe peptic ulcer disease was present.

Data on pre-endoscopic management (PPI dose and duration, prior non-invasive *H. pylori* testing, and symptom duration) were not systematically recorded in the electronic medical record and therefore were not included in the analysis.

### 2.3. Data Analysis and Outcome Assessment

Endoscopic yield was analyzed according to age, sex, and ethnicity. Age-related outcomes were evaluated using predefined thresholds for gastroscopy as recommended by international guidelines. These age thresholds were interpreted in the context of the American College of Gastroenterology/Canadian Association of Gastroenterology (ACG/CAG) guideline, which recommends considering endoscopy in patients aged ≥60 years, and the NICE guideline, which advises endoscopy primarily in patients aged ≥55 years. The impact of demographic and clinical factors on the detection of significant pathology was further evaluated, with specific emphasis on identifying independent predictors of gastrointestinal malignancy, precancerous lesions, and clinically significant findings (CSFs). The latter category encompassed malignancies, precancerous conditions, severe gastritis or esophagitis, and PUD.

To determine the optimal age threshold for predicting gastrointestinal malignancy and clinically significant pathology, ROC curve analysis was performed. The area under the curve (AUC) was used to assess the discriminatory performance of age as a predictive factor, with sensitivity and specificity analyzed across different age cutoffs.

### 2.4. Statistical Analysis

Continuous variables were summarized as means with standard deviations, and categorical variables as frequencies and percentages. Subgroup comparisons were performed using Student’s t-test for normally distributed continuous variables or the Mann–Whitney U test for non-parametric data. Categorical variables were compared using the chi-square test, with Fisher’s exact test applied when expected cell counts were small. Missing data were minimal (~1.5%), and a complete-case analysis was therefore undertaken. Variables associated with outcomes in univariate analyses were entered into multivariable logistic regression models to identify independent predictors of malignancy, precancerous lesions, and clinically CSFs. Receiver operating characteristic (ROC) curve analysis was used to determine optimal age cutoffs for malignancy and CSF. All statistical tests were two-sided, with statistical significance set at *p* < 0. Analyses were performed using SPSS version 25 (IBM Corp., Armonk, NY, USA).

## 3. Results

A total of 16,129 gastroscopy reports performed during the study period for various indications were reviewed, of which 3090 procedures were conducted for the evaluation of dyspepsia. After applying exclusion criteria, 3022 patients were included in the final analysis. Among these, 321 (10.6%) underwent gastroscopy for dyspepsia in combination with alarm symptoms, including anemia, weight loss, persistent vomiting, or dysphagia. Gastric biopsies were obtained in the majority of cases (77.9%), while a rapid urease test was performed in 28.3% of procedures. The mean patient age was 51.7 ± 18.3 years (range, 18–95 years), with a slight female predominance (56%). The Jewish population comprised 79.1% of the cohort, mirroring its proportion in the general population.

### 3.1. Endoscopic and Histologic Findings

Almost 90% of patients received an endoscopic diagnosis of gastritis. Given the limited correlation between visual appearance and histologic inflammation, this finding should be interpreted with caution, as endoscopy may overestimate true histologic gastritis.

Biopsies were obtained in 2356 patients (77.9% of the cohort). Among these, 443 (18.8%) had normal histologic findings, 502 (21.3%) had non-specified chronic gastritis, and 351 (14.9%) had *H. pylori*–associated gastritis.

Urease testing was performed in 855 patients (28.3%), of whom 120 (14%) had a negative result. Across the entire cohort, *H. pylori* gastritis was identified in 1066 patients (35.3%), based on either histology or urease testing, with unique cases counted to avoid duplication.

Based on endoscopists’ impressions, 81.1% of endoscopic gastritis cases were classified as mild. The most frequent endoscopic gastritis phenotypes were erythematous/superficial gastritis (48.2%), erosive gastritis (27.1%), nodular gastritis (21.1%), and atrophic gastritis (2.3%). Gastritis was confined to the distal stomach or antrum in 71.2% of cases, whereas pangastritis was observed in 11.1%.

The prevalence of FD, defined by the absence of clinically significant endoscopic or histologic abnormalities, was 55.9%. Other notable findings included hiatal hernia (16.4%), reflux esophagitis (7.8%), and Barrett’s esophagus (0.53%). The majority of reflux esophagitis cases were mild, with only 6.3% presenting with Los Angeles grade C-D esophagitis. Severe PUD, characterized by gastric or duodenal ulcers, was identified in 2.5% of patients.

Precancerous gastric lesions, including atrophic gastritis, intestinal metaplasia, and dysplasia, were detected in 12.8% of biopsy specimens. The overall malignancy rate was low (0.79%), consisting of seven cases of gastric lymphoma, three cases of esophageal carcinoma, and fourteen cases of gastric carcinoma. Notably, over 70% of malignancies were diagnosed in patients with alarm symptoms.

### 3.2. Age-Based Analysis and ROC Assessment

To assess the diagnostic utility of age, patients were stratified into <50 years (*n* = 1217) and ≥50 years (*n* = 1805) (Table 1), and all percentages were calculated within each age group. Patients aged ≥50 years had a significantly higher prevalence of precancerous gastric lesions (13.5% vs. 4.8%; *p* < 0.0001) and gastric cancer (0.7% vs. 0.2%; *p* = 0.056) compared with those <50 years. When all upper gastrointestinal malignancies were considered (gastric, esophageal, and lymphoma), the malignancy rate was 1.16% in patients ≥50 years versus 0.24% in those <50 years (*p* = 0.04). Only three malignancies were identified in patients younger than 50 years, and all occurred in the presence of alarm symptoms.

In contrast, *H. pylori*–related gastritis (defined by positive histology and/or urease testing) was more frequent in younger patients: 43.0% of those <50 years versus 30.0% of those ≥50 years (*p* < 0.0001), underscoring the different etiologic profile of dyspepsia across age strata.

ROC analysis was performed to evaluate the discriminatory ability of age for predicting CSFs and malignancy. For CSFs, age demonstrated moderate discrimination, with an area under the curve (AUC) of 0.65, and the Youden-derived optimal cutoff was 50 years. At this threshold, the estimated sensitivity was high (≈79%), whereas specificity was modest (≈43%), consistent with a strong negative predictive value (≈93%) but a relatively low positive predictive value (≈18%). For malignancy, age exhibited better discriminatory performance, yielding an AUC of 0.72, with an optimal cutoff of 54 years. This threshold provided high sensitivity (≈88%) and excellent negative predictive value (≈99.8%), though specificity remained limited (≈41%) given the low overall prevalence of cancer.

### 3.3. Ethnicity-Based Analysis

Ethnic differences were evident across baseline characteristics and several endoscopic and histologic outcomes (Table 2). Arab patients presented for evaluation at a substantially younger age than Jewish patients (45.1 ± 18.5 vs. 54.3 ± 17.96 years, *p* < 0.0001) and exhibited a lower proportion of females (51% vs. 57%, *p* = 0.002). The overall prevalence of *H. pylori* gastritis—defined by positive biopsy or urease testing—was markedly higher among Arab patients (approximately 48% vs. 32%, *p* < 0.0001). In contrast, precancerous gastric lesions were significantly less common in the Arab group (7.4% vs. 10.7%, *p* = 0.017). Despite these differences, rates of endoscopic gastritis and reflux esophagitis were broadly comparable between the two populations.

### 3.4. Multivariate Analysis of Predictors of CSF

Multivariate logistic regression identified age over 50 years (OR = 4.89, *p* = 0.036, 95% CI: 1.109–21.602) and the presence of alarm symptoms (OR = 31.421, *p* < 0.0001, 95% CI: 10.264–96.192) as independent predictors of malignancy (Table 3). Conversely, sex and ethnicity were not significant independent predictors of cancer risk. A total of 24 malignancies were available for inclusion in this multivariable model.

CSFs were independently associated with age over 50 years (OR = 2.590, *p* < 0.0001, 95% CI: 2.019–3.324), male sex (OR = 1.404, *p* = 0.002, 95% CI: 1.135–1.737), and alarm symptoms (OR = 1.793, *p* < 0.0001, 95% CI: 1.334–2.410).

For precancerous gastric lesions, age over 50 years remained the strongest predictor (OR = 3.053, *p* < 0.0001, 95% CI: 2.260–4.124), while male sex and alarm symptoms were not independently associated with an increased risk.

## 4. Discussion

In this large, real-world cohort of dyspeptic patients referred for gastroscopy, we systematically characterized demographic, clinical, endoscopic, and histologic determinants of CSFs. The study offers a comprehensive risk profile by quantifying age- and symptom-related associations, examining ethnic variation within a unified healthcare system, and evaluating not only malignancy but also the wider spectrum of precancerous and clinically relevant gastric pathology. Dyspepsia accounted for nearly one-fifth of all gastroscopy referrals during the study period, underscoring its substantial impact on endoscopy services. Consistent with prior epidemiologic data, we observed a modest female predominance among dyspeptic patients [21,22,23]. Arab patients presented at a significantly younger age than Jewish patients, and more than 40% of procedures were performed in individuals younger than 50 years, with the proportion approaching 60% in the Arab subgroup. This pattern mirrors reports from other low-to-intermediate gastric cancer incidence regions, where most endoscopies in dyspeptic patients reveal benign pathology—typically gastritis, peptic ulcer disease, or functional dyspepsia—with very low malignancy rates [24]. Together, these findings raise important questions regarding appropriateness of referral and the efficiency of current dyspepsia work-up pathways.

A key observation in our cohort was the high prevalence of *H. pylori*–associated gastritis in patients under 50 years, despite guideline recommendations that emphasize non-invasive testing and empiric acid suppression as first-line strategies in low-risk dyspepsia [25]. Although most patients had been empirically treated with PPIs before endoscopy, the high rate of *H. pylori*–positive gastritis in younger individuals suggests that endoscopy is often used prematurely, rather than after a structured “test-and-treat” strategy. Our data therefore support the need for enhanced education and standardized pathways in primary care to encourage non-invasive *H. pylori* testing and adequately dosed, time-limited PPI trials before endoscopic referral, especially in patients under 50 years without alarm symptoms [26,27]. Such an approach has the potential to reduce low-yield procedures and associated healthcare costs while preserving patient safety.

The overall prevalence of upper gastrointestinal malignancy in dyspeptic patients in our cohort was below 1% and did not differ materially from that of the general gastroscopy population, indicating that dyspepsia per se is a weak marker of cancer risk. This aligns with large endoscopy database studies from Asia and the Middle East, which have similarly reported very low malignancy rates among dyspeptic patients, particularly at younger ages [28,29]. However, within this low-risk context, alarm symptoms retained important discriminatory value: the odds of malignancy were markedly higher in patients presenting with alarm features, even though this subgroup constituted only about one in ten dyspeptic referrals. These findings complement previous work showing that age and alarm symptoms have only modest predictive performance when considered in isolation [30,31], but our results suggest that in a real-world setting, alarm features still identify a small, higher-risk subset in whom endoscopy is clearly justified.

Our age-stratified analyses and ROC-based assessment offer further refinement of risk. Cancer detection was approximately fivefold higher in patients aged ≥50 years compared with those <50 years, and all malignancies in younger patients occurred in the presence of alarm symptoms. ROC analysis yielded an AUC of 0.72 for malignancy, with an optimal age threshold of 54 years, and an AUC of 0.65 for CSFs with a threshold of 50 years. These values indicate moderate discrimination: age alone is an informative but imperfect predictor that must be interpreted alongside clinical context. Importantly, at the chosen cutoffs, sensitivity and negative predictive value for malignancy were high, whereas specificity was modest, meaning that lower age thresholds preferentially enhance safety (few missed cancers) at the cost of additional low-yield procedures. Viewed in a broader regional and global context, the present results are consistent with contemporary studies, where malignancy detection in dyspeptic populations remains rare and where both age and alarm features have shown limited precision as independent predictors of clinically significant disease [24,28,32]. Together, these data suggest that rigid transplantation of a single global age cutoff is unlikely to be optimal; rather, thresholds may need to be calibrated to regional cancer incidence, resource constraints, and local patterns of *H. pylori* and precancerous disease.

By extending our analysis beyond overt malignancy to include CSFs and precancerous gastric lesions, we show that age ≥ 50 years also enriches for clinically meaningful pathology short of cancer. Age ≥ 50 years, male sex, and alarm symptoms were independent predictors of CSFs in multivariable models, and age ≥ 50 years was the only independent predictor of precancerous gastric conditions. Although some histologic abnormalities such as focal atrophic gastritis may carry uncertain long-term risk, others, including intestinal metaplasia and gastric dysplasia, are well-established precursors of gastric cancer and form the basis of surveillance recommendations in Western and Asian guidelines [33]. Our data therefore provide additional support for age-based risk stratification that considers both immediate cancer yield and the detection of lesions that warrant long-term endoscopic follow-up.

Ethnicity showed a more complex pattern. In unadjusted analyses, Arab patients had higher *H. pylori* prevalence and lower rates of precancerous conditions than Jewish patients, reflecting different background risk profiles within the same healthcare system. However, after adjustment for age, sex, and alarm symptoms, ethnicity was not an independent predictor of malignancy, CSFs, or precancerous lesions. This suggests that observed ethnic differences are largely mediated through age and infection-related factors rather than ethnicity per se. Nonetheless, the divergent patterns of *H. pylori* infection and precancerous histology highlight the importance of region- and subgroup-specific data to inform tailored screening and surveillance strategies [34].

Several limitations merit consideration. First, this was a single-center, retrospective study, which may limit generalizability beyond our catchment area. Second, although our cohort reflects routine practice in a large, university-affiliated hospital, key aspects of pre-endoscopic management such as PPI dose and duration, non-invasive *H. pylori* testing, and symptom duration, were inconsistently documented and therefore could not be analyzed in detail. Information on dietary factors, smoking, and medication adherence was likewise incomplete, constraining a more nuanced assessment of referral appropriateness, particularly in patients younger than 50 years. Third, despite adjustment for baseline demographic and clinical differences between ethnic groups, the risk of residual confounding cannot be excluded given the unequal group sizes and observational design. In addition, the multivariable malignancy model included only 24 events, which inevitably limits model stability and raises the possibility of overfitting despite careful variable selection. These issues argue that our age-related cancer risk estimates and ROC-derived thresholds should be viewed as exploratory and hypothesis-generating rather than definitive.

Notwithstanding these limitations, the strengths of this study include its large sample size, systematic capture of endoscopic and histologic data, explicit consideration of precancerous conditions and CSFs as outcomes, and the ability to explore ethnic and age-related differences within a single, integrated healthcare system. Taken together, our findings support a selective, risk-stratified approach to endoscopy in dyspepsia, in which age (particularly ≥50 years) and alarm symptoms remain central triage variables, but are embedded within a broader strategy that prioritizes non-invasive management in low-risk patients and acknowledges regional variation in gastric cancer epidemiology and healthcare capacity.

## Figures and Tables

**Table 1:** Comparison of baseline characteristics and major outcomes of age groups.

Age Group	Age < 50; *n* = 1217	Age ≥ 50; *n* = 1805	*p* Value
Age mean + SD	32.8 ± 10.65	64.5 ± 9.7	N/A
Gender			*p* = 0.26
Male	551 (45.3%)	779 (43.2%)	
Female	666 (54.7%)	1026 (56.8%)	
Race			*p* < 0.0001
Jews	839 (68.9%)	1552 (86.0%)	
Arabs	378 (31.1%)	253 (14.0%)	
Endoscopic diagnosis			
Gastritis	1017 (83.6%)	1604 (88.9%)	*p* = 1.00
Duodenitis	177 (14.5%)	261 (14.5%)	*p* = 0.96
Reflux esophagitis	90 (7.4%)	127 (7.0%)	*p* = 0.72
Urease test			*p* = 0.007
Performed	457 (37.6%)	398 (22%)	
Positive (of performed)	392 (85.8%)	343 (86.2%)	
Alarm symptoms			*p* = 0.40
Dyspepsia	1095 (90%)	1606 (89%)	
Dyspepsia and alarm	122 (10%)	199 (11%)	
Gastritis type			*p* < 0.0001
Not specified	8 (1%)	14 (1%)	
Superficial	558 (46%)	889 (49%)	
Erosive	241 (20%)	572 (32%)	
Nodular	394 (32%)	242 (13%)	
Atrophic	10 (1%)	60 (3%)	
Other	6 (0.5%)	28 (2%)	
Gastritis Severity			*p* = 0.86
Mild	977 (80%)	1457 (81%)	
Moderate	230 (19%)	328 (18%)	
Severe	7 (1%)	9 (1%)	
Gastritis location			*p* = 0.44
Antrum/Distal	874 (77%)	1263 (75%)	
Corpus/Proximal	136 (12%)	213 (13%)	
Pan-gastritis	130 (11%)	216 (13%)	
Reflux esophagitis grade			*p* = 0.44
A *n* (%)	63 (64%)	84 (62%)	
B *n* (%)	32 (32%)	41 (30%)	
C-D *n* (%)	4 (4%)	11 (8%)	
Gastric Cancer	2 (0.2%)	12 (0.7%)	*p* = 0.056
pre-cancerous lesions	58 (4.8%)	244 (13.5%)	*p* < 0.0001
Esophageal cancer	0	3 (0.2%)	*p* = 0.28
Gastric lymphoma	1 (0.1%)	6 (0.3%)	*p* = 0.25

**Table 2:** Comparison of baseline characteristics and main outcomes of ethnicity groups.

Age Group	Jews; *n* = 2391	Arabs; *n* = 631	*p* Value
Age mean + SD	54.27 ± 17.96	45.06 ± 18.51	*p* < 0.0001
Gender			*p* = 0.002
Male	1018 (42.6%)	312 (49.4%)	
Female	1373 (57.4%)	319 (50.6%)	
Endoscopic diagnosis			
Gastritis	2065 (86.4%)	556 (88.1%)	*p* = 1.00
Duodenitis	331 (13.8%)	107 (17.0%)	*p* = 0.056
Reflux esophagitis	177 (7.4%)	38 (6.0%)	*p* = 0.72
Alarm symptoms			*p* = 0.83
Dyspepsia	2135 (89%)	566 (90%)	
Dyspepsia and alarm	256 (11%)	65 (10%)	
Gastritis type			*p* < 0.0001
Not specified	17 (1%)	5 (1%)	
Superficial	1209 (51%)	238 (38%)	
Erosive	652 (27%)	161 (25.5%)	
Nodular	424 (18%)	212 (34%)	
Atrophic	63 (3%)	7 (1%)	
Other	26 (1%)	8 (1%)	
Gastritis Severity			*p* = 0.004
Mild	1954 (82%)	480 (76%)	
Moderate	413 (17%)	145 (23%)	
Severe	12 (0.5%)	4 (1%)	
Gastritis location			*p* = 0.041
Antrum/Distal	1671 (75%)	466 (79%)	
Corpus/Proximal	293 (13%)	56 (9.5%)	
Pan-gastritis	278 (12%)	68 (11.5%)	
Reflux esophagitis grade			
A *n* (%)	125/189 (66)	22/46 (48%)	
B *n* (%)	54/189 (28.5)	19 (41%)	
C-D *n* (%)	10 (5.1%)	5 (10%)	
Gastric Cancer	13 (0.5%)	1 (0.2%)	*p* = 0.32
Gastric pre-cancerous lesions	255 (10.7%)	47 (7.4%)	*p* = 0.017
Esophageal cancer	3 (0.1%)	0	*p* = 1.00
Gastric lymphoma	7 (0.3%)	0	*p* = 0.36

**Table 3:** Predictors of outcome. A multivariate analysis.

Cancer Predictors—A Multivariate Analysis				
Variable	Odds Ratio	95% Confidence Interval		*p*-Value
		Lower	Upper	
Age over 50	4.894	1.109	21.602	0.036
Sex (male)	1.317	0.513	3.380	0.567
Ethnicity (Jews)	3.604	0.468	27.781	0.219
Alarm symptoms	31.421	10.264	96.192	<0.0001
Clinically significant findings predictors—A multivariate analysis				
Age over 50	2.590	2.019	3.324	<0.0001
Sex (male)	1.404	1.135	1.737	0.002
Ethnicity (Jews)	1.148	0.863	1.528	0.343
Alarm symptoms	1.793	1.334	2.410	<0.0001
Precancerous gastric conditions predictors—A multivariate analysis				
Age over 50	3.053	2.260	4.124	<0.0001
Sex (male)	1.157	0.908	1.475	0.238
Ethnicity (Jews)	1.172	0.840	1.636	0.350
Alarm symptoms	1.108	0.762	1.612	0.592

## Data Availability

The data underlying this study are not publicly available due to institutional and ethical restrictions related to patient privacy. De-identified data may be made available from the corresponding author upon reasonable request and with approval from the institutional ethics committee.
